# Implementing Guidelines for hypothermia prevention with Local adaptation to keep periOperative patients Warm (GLOW): protocol of a stepped-wedge cluster randomised hybrid type II effectiveness-implementation study

**DOI:** 10.1136/bmjopen-2024-091577

**Published:** 2025-02-12

**Authors:** Judy Munday, Jed Duff, Fiona M Wood, David Sturgess, Samantha Keogh, Nicholas Ralph, Nicole M White, Hannah Carter, Ian D Graham

**Affiliations:** 1 School of Health, University of The Sunshine Coast, Sippy Downs, Queensland, Australia; 2 Faculty of Health and Sports Sciences, University of Agder, Kristiansand, Vest-Agder, Norway; 3 School of Nursing/Centre for Healthcare Transformation, Queensland University of Technology, Brisbane, Queensland, Australia; 4 Nursing and Midwifery Research Centre, Royal Brisbane and Women's Hospital, Herston, Queensland, Australia; 5 School of Biomedical Sciences, The University of Western Australia, Perth, Western Australia, Australia; 6 Burns Service of Western Australia, Government of Western Australia Department of Health, Perth, Western Australia, Australia; 7 PA-Southside Clinical Unit, Faculty of Medicine, The University of Queensland, Brisbane, Queensland, Australia; 8 The University of Queensland, Brisbane, Queensland, Australia; 9 Australian Centre for Health Services Innovation, School of Public Health and Social Work, Queensland University of Technology, Brisbane, Queensland, Australia; 10 School of Epidemiology and Public Health, University of Ottawa, Ottawa, Ontario, Canada

**Keywords:** SURGERY, Nursing Care, ANAESTHETICS, Clinical Trial, Implementation Science

## Abstract

**Introduction:**

Perioperative hypothermia is a common and preventable complication of surgery. Systems level change that enables perioperative teams to integrate hypothermia prevention into practice in ways that are contextually appropriate is needed. The purpose of this trial is to evaluate the effectiveness and implementation of perioperative hypothermia prevention guidance with local adaptation on clinical, implementation and economic outcomes. Our objective is to decrease the risk of patients developing perioperative hypothermia.

**Methods and analysis:**

A hybrid type II effectiveness-implementation study. An incomplete stepped-wedge cluster randomised controlled trial design will be used, with a 6-month transition period for implementation. Perioperative departments from five major public hospitals in South East Queensland, Australia, will participate over 27 months. The co-primary outcomes are (effectiveness) hypothermia on arrival to the post anaesthetic care unit (PACU) and (implementation) extent of temperature monitoring and active warming. Secondary clinical effectiveness outcomes include hypothermia at any perioperative time point, PACU and hospital length of stay, intraoperative or post-anaesthetic adverse events, blood transfusions and surgical site infection. Secondary implementation outcomes include pre-transition measures of adoptability and implementability, and post-transition measures of adoption, fidelity of implementation strategy and site team learning. Cost-effectiveness will evaluate implementation costs and quality-adjusted life years. Based on the number and unequal sizes of clusters, we used a constrained randomisation approach for sample size determination to minimise the imbalance between control and intervention period sample sizes. Generalised linear mixed models will be used to analyse primary and secondary outcomes.

**Ethics and dissemination:**

Ethical approval was obtained with a waiver of consent to access clinical records (reference: HREC/2023/MNHB/94571). Informed consent will be sought from patients completing surveys. Consent will be implied from clinicians participating in implementation evaluation. Findings will be disseminated through journal publications and conference presentations. Practice and policy recommendations will be collaboratively developed with partner organisations.

**Trial registration number:**

Prospective registration of the trial was obtained on 28 July 2023 on the Australia and New Zealand Clinical Trials Registry Network: ACTRN12623000814673.

**STRENGTHS AND LIMITATIONS OF THIS STUDY**

- This effectiveness-implementation study will evaluate both (1) consensus-based simple principles and recommendations for perioperative hypothermia prevention and (2) a team-based implementation approach with local adaptation.
- A stepped-wedge cluster trial design allows all hospital sites to begin implementation at different times, with each site acting as its own control.
- An external–internal facilitation model will support interdisciplinary team-based implementation at each site, guided by the Knowledge-to-Action framework.
- We use a pragmatic design for data collection; hence outcome evaluation relies on completeness of patient and hospital data sets.

## Introduction

 Perioperative systems of care are not designed to keep patients warm. General and neuraxial anaesthesia impairs thermoregulation, meaning heat loss is inevitable without warming.^1^ Patients have limited control over exposure to cold ambient conditions and their thermal comfort. Body temperature is not consistently monitored throughout perioperative care.^2^ Most temperature monitoring and warming occur after surgery is complete^2^ and after patients have already been exposed to the complications of hypothermia.^2 3^ Warming practices are often not proactive or consistently based on patient needs.^2^ Indeed, pre-emptive and coordinated hypothermia prevention practices may be constrained in a system where competing priorities include patient flow and productivity.^4^

The discomfort of perioperative hypothermia and associated shivering is remembered by patients well after surgery.^1^ The potential adverse outcomes reported in the literature are wide-ranging, including increased wound infection,^5^,^7^ risk of surgical bleeding^8^ and blood transfusions,^5^ myocardial injury and morbid cardiac events,^8 9^ delays in drug metabolism,^1^ prolonged post-anaesthetic recovery times and hospital stays.^7 10^ The cost of perioperative hypothermia to the healthcare system is an estimated $A1.2 billion per annum.^11^ Prevention would produce an estimated net cost-benefit of $A689 659 per annum for an average size healthcare facility, due to the prevention of hypothermia-related morbidity.^12^

While multiple comprehensive clinical guidelines for perioperative hypothermia exist, there has been little impact on practice.^13 14^ The majority of guidance has been developed by discipline-specific organisations and colleges, rather than building consensus across all professions. Hypothermia prevention is a complex intervention, with interdependent activities such as temperature monitoring and warming. However, the existing implementation research on hypothermia prevention is extremely limited by study size, scope and quality. Efforts to implement complex, multi-component guidelines and pathways in other aspects of perioperative care have resulted in little change in practice or improvement in patient outcomes.^15^,^17^ Findings from the Enhanced Peri-Operative Care for High-risk patients trial^16^ show that extensive checklists and pathways can stall progress in improving patient care. Adaptability is essential when designing quality improvement across different contexts.

Rather than adding to existing hypothermia guidelines, we sought to develop simple principles with practice recommendations for perioperative prevention that provide direction for practice, while allowing for local adaptation and implementation.^18^ Working with simple principles accounts for the complexity of care and supports frontline teams in developing context-specific change strategies.^19^ If transformation in this context is to be realised, then different and highly tailored solutions will be required. Uniform guidelines and single solutions are unlikely to work, meaning implementation needs to accommodate a highly contextualised approach.

## Methods and analysis

### Aims

The aims of this trial are to evaluate the effectiveness and implementation of perioperative hypothermia prevention guidance with local adaptation on clinical, implementation and economic outcomes. The co-primary aims reflect a hybrid type 2 effectiveness-implementation study.^20^ A hybrid type 2 study has the dual focus of testing both interventions and implementation strategies simultaneously.^20^

### Study design and setting

**Figure 1:**
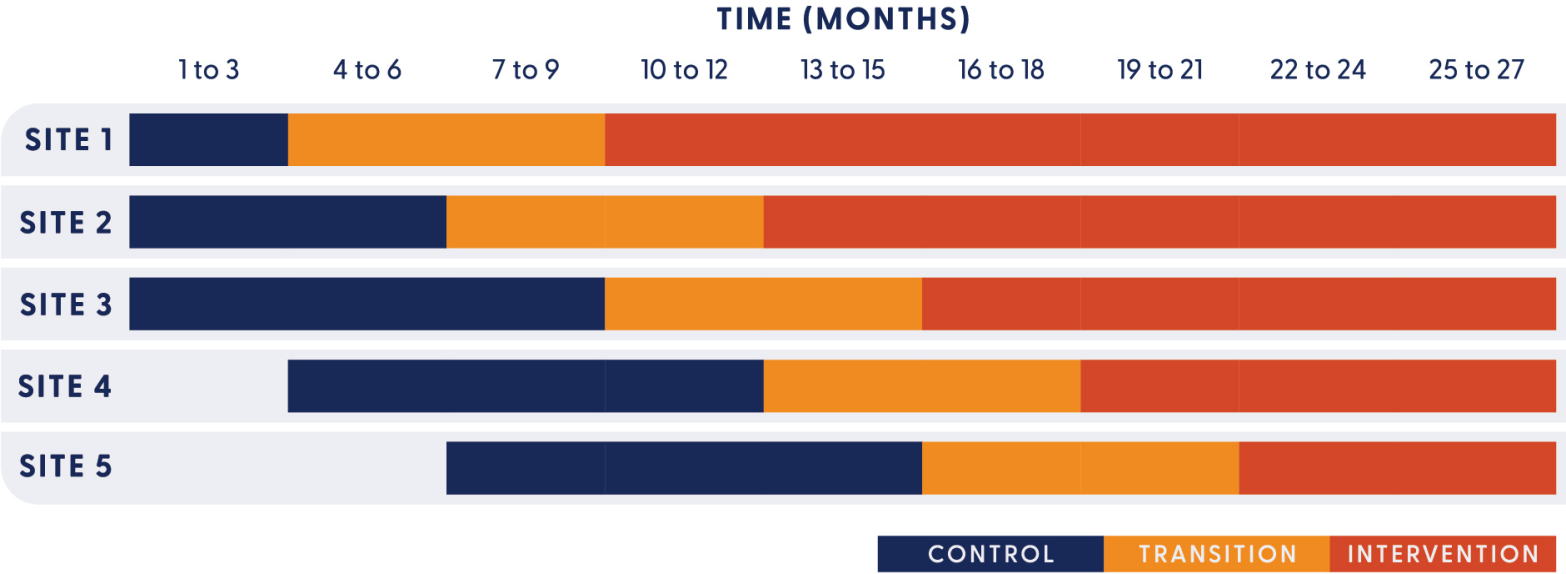
Stepped-wedge design.

An incomplete stepped-wedge cluster randomised controlled trial design will address both aims. Stepped-wedge cluster designs allow all sites to begin implementation at different times, with each site acting as its own control.^21^ Figure 1 presents the trial design. Perioperative departments from five public hospitals in South East Queensland, Australia, will participate in the trial over 27 months (November 2023 to February 2026): three metropolitan hospitals (sized from 323 to 1054 beds) and two regional hospitals (from 438 to 738 beds). All hospitals provide elective and emergency surgery. Surgical specialties provided by sites include general surgery, orthopaedics, gynaecology, plastics and reconstructive, vascular, urology, maxillofacial, ear, nose and throat, ophthalmology. Two of the metropolitan hospitals provide neurosurgery, and one of these also provides specialist burns care. A random sample of patients undergoing surgery during the control or intervention period at each hospital will be included in the analysis. This protocol is reported according to the Standard Protocol Items: Recommendations for Interventional Trials statement^22^ and with reference to guidance for design and reporting of stepped-wedge cluster randomised controlled trials.^21^

### Intervention

The Knowledge-to-Action (KTA) framework provides a structure for planned action and implementation.^23 24^ The framework emphasises the importance of adaptation of research evidence to the local context in planned practice change.^25^ Our creation of a knowledge tool in the form of consensus-based simple principles and recommendations was situated within the Knowledge Creation process.^23^ The KTA Action Cycle^23^ is used to guide the process of practice change undertaken by teams at each site.

#### Consensus-based simple principles and recommendations

Using a consensus-based process reported previously in this journal,^18^ the evidence-based simple principles and accompanying detailed recommendations for hypothermia prevention, were adapted for the Australian context. The three principles for perioperative hypothermia prevention are (1) actively monitor core temperature for all patients at all times, (2) warm actively to keep temperature above 36°C and patients comfortable and (3) minimise exposure to cold at all stages of perioperative care.^18^ These represent the three main strands of hypothermia prevention, providing a guide to action with the support of detailed recommendations where needed. Rather than trying to enforce compliance with detailed guidelines, working with principles is compatible with our tailored implementation approach.^12 19^

#### Implementation strategy

**Figure 2:**
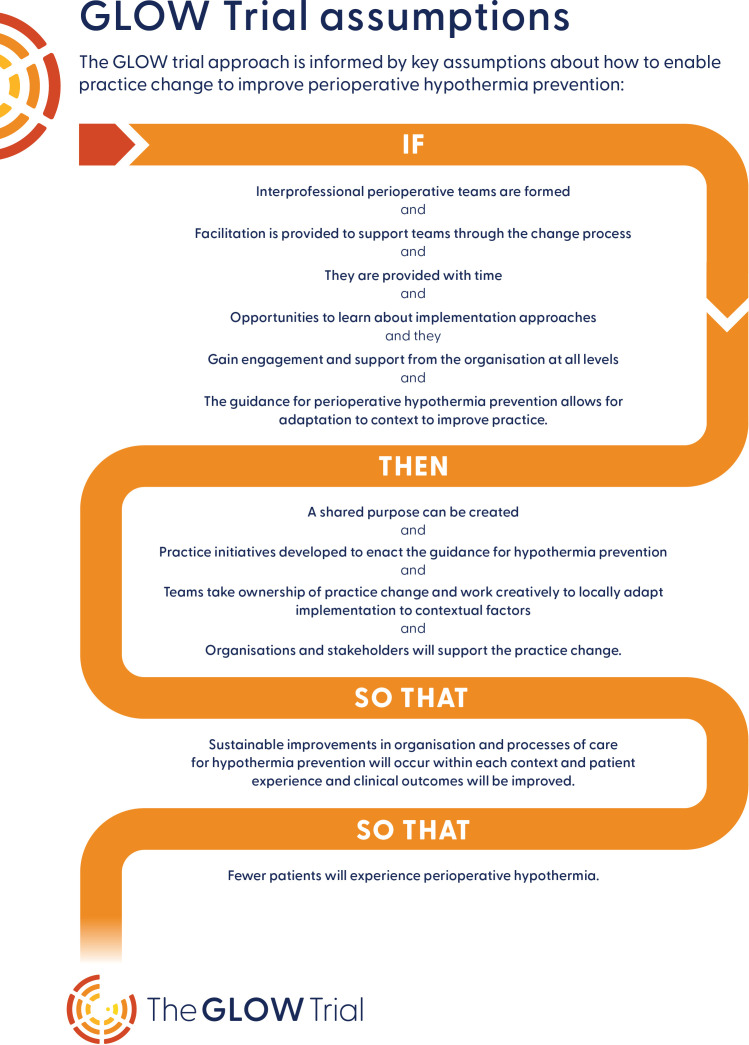
GLOW trial assumptions. GLOW, Guidelines for hypothermia prevention with Local adaptation to keep periOperative patients Warm.

The core components of our implementation strategy include: (1) team-based implementation with local adaptation; (2) external–internal facilitation. Our assumptions about how we will enable practice change to improve hypothermia prevention are presented in figure 2.

##### Local Site Implementation Teams

Site Implementation Teams (or GLOW teams) will be formed at each hospital site to lead local implementation, supported by internal and external facilitation. Site Implementation Teams will aim to include members from each main professional group involved in the care of patients in the perioperative setting, who have a role in perioperative hypothermia prevention and who have influence among colleagues in either leadership or practice positions. With facilitation support, Site Implementation Teams will dedicate time to local implementation of the simple principles guided by the KTA Action Cycle.^23^ Site Implementation Teams will determine the need for specific practice change initiatives to address knowledge-practice gaps for perioperative hypothermia prevention in their context, in alignment with the simple principles. Teams will work through the Action Cycle, including small-scale testing of practice change initiatives, selection and tailoring of implementation strategies, monitoring of uptake, evaluation of patient and organisational outcomes and planning for sustainment.^23 25^

##### External–internal facilitation

External–internal facilitation models have enabled large-scale transformation by working with frontline acute care teams.^26^ In the context of implementation, facilitation is both a role and a process that enables purposeful action to achieve practice change.^27^ External facilitation will be provided by the trial team and will support a trial-funded internal facilitator (0.6 full-time equivalent). Internal facilitators are registered nurses recruited from within each perioperative department who demonstrate an interest in perioperative hypothermia prevention, and who are well-known and influential within the department. In turn, each Site Implementation Team will receive support throughout the 6-month transition period (see figure 1) from both the internal and external facilitators as they apply the KTA Action Cycle.^23^ Facilitation of participatory group decision-making both within the site teams and progressively in each perioperative department will build ownership of practice change.^26^

Site Implementation Teams are provided with a practical workbook as a written resource to guide implementation of the simple principles using the KTA framework.^23^ An online MS Teams site for each Site Implementation Team will contain stand-alone resources such as Plan-Do-Study-Act^28^ planners, action plans and audit tools that can be adapted for each site in addition to providing a channel for team communication and collaboration.

### Participants: inclusion and eligibility criteria

The patient study population will meet the inclusion criteria and undergo surgery at the hospital sites during the study period. A subset of patients meeting the inclusion criteria will also be invited to participate in data collection of secondary patient-reported outcomes (PROs) for inclusion in the economic analysis.

Patients’ eligibility criteria are: (1) adults aged 16 years or older; (2) having undergone general or neuraxial anaesthesia, elective or emergency surgery at the hospital site during the site’s control or intervention period. Exclusion criteria are: (1) patients receiving planned therapeutic hypothermia; (2) undergoing local anaesthesia or sedation only, or local anaesthesia with sedation only; (3) patient transfer directly to the intensive care unit from operating theatres; (4) cardiac or obstetric surgery.

Clinicians involved in Site Implementation Teams (registered or enrolled nurses, anaesthetic technicians, surgical and anaesthetic medical personnel) will be eligible to complete specific implementation strategy evaluation measures. Additionally, clinicians who have worked in the perioperative department during the trial will also be approached to complete implementation evaluation surveys.

#### Recruitment

Data collection will occur without direct recruitment of patients, using patient and hospital records with waiver of consent approved by hospital and university ethics committees and with Public Health Act approval. Direct recruitment of patients with informed consent will only be undertaken for the subset of patients invited to participate in data collection required for PROs for inclusion in the economic analysis. Using standard preoperative information methods at each hospital, a study flyer will be provided prior to surgery with contact details of the trial team and a QR code link to the participant information. Informed consent can be provided via hard copy or online consent form, depending on each participant’s preference. The surveys will also be provided via the participant’s preferred mode of contact (via email or text message). Patients who consent to participation in data collection of PROs will be able to withdraw their data at any time up until the point of de-identification, which occurs after the completion of their final survey.

Implied consent will be assumed for clinicians completing the implementation evaluation surveys, including clinicians who are members of Site Implementation Teams. A participant information form for clinicians will be provided with electronic or hard copy survey. Clinicians who participate in implementation evaluation surveys can withdraw their consent until survey submission, at which point data is non-identifiable.

#### Screening of participants

A monthly list of patient identification numbers and surgery details (date and time) will be provided by Health Information Services at each hospital and patients will be screened for eligibility before random sampling. For the recruitment of patients for PRO surveys, prospective screening will be undertaken to ensure only patients meeting inclusion criteria will be invited to complete the surveys. Participants accessing online participant information will be asked screening questions before proceeding to the participant information and consent form. Participants accessing hard copy participant information and/or completing hard copy consent forms will be screened for eligibility by preadmission staff.

### Randomisation

Randomisation is defined at the site level, meaning that a cluster is defined by one hospital site. Each site will be subject to a variable control and intervention period, based on the randomisation order (figure 1). All sites will go through a fixed 6-month transition period at the end of their control period. During the control period, clusters continue with normal care. During the 6-month transition period, implementation by Site Implementation Teams will commence. The only data collected during the transition will be locally collected data by Site Implementation Teams used for implementation planning purposes. Sites will then switch to and remain in the intervention period of data collection until the end of the trial, between 6 and 18 months depending on randomisation order. To reduce data collection burden while maintaining adequate statistical power, an incomplete design will be used. This means that the sites randomised to clusters 1–3 will commence their control period in month 1. Clusters 4 and 5 will commence their control period in months 4 and 7, respectively.

#### Sequence generation

The randomisation of sites to treatment sequences in figure 1 will result in each hospital site being randomised to begin implementation and commence the transition period at a different time.^21^ Sequence generation will be conducted by a statistician not involved in the trial outcome analysis. We will use a constrained randomisation approach to account for facility size, which offers a pragmatic yet statistically robust approach for evaluating complex interventions across multiple study sites of varying surgical capacity.^29 30^

### Allocation concealment

Site randomisation to clusters 1–5 will occur only after all sites have been enrolled. Only the chief investigators and project coordinator will have access to the cluster randomisation order. Allocation concealment will be maintained until 12 weeks prior to a cluster commencing implementation, to allow for recruitment of internal facilitators and for Site Implementation Teams to be formed and prepared for the implementation transition period.^21^

### Blinding

Due to the nature of the intervention and trial design, blinding healthcare professionals, outcome evaluators and the trial statistician is not possible. We will attempt to mask the primary outcome from outcome evaluators. All patients will be masked during the intervention period.

### Outcome measures

Table 1 presents primary, secondary and other outcomes including measurement variables and time points.

**Table 1:** Outcome evaluation

	Outcome variable	Measure
Clinical effectiveness outcomes		
1	Perioperative hypothermia on post anaesthetic care unit (PACU) arrival	Body temperature <36°C recorded within 15 min of PACU arrival time
2	Perioperative hypothermia at any time point	Body temperature <36°C at any time point: (1) within hour prior to surgery; (2) on induction; (3) intraoperatively; (4) during PACU admission; (5) on ready to discharge from PACU
3	Hospital length of stay	Days of separation (hospital)
4	PACU length of stay	Minutes (from PACU admission to discharge)
5	Intraoperative events	Complications recorded during the surgical episode
6	PACU events	Complications recorded during PACU admission
7	Blood product transfusions	Number of units transfused in surgery and PACU
8	Surgical site infection (SSI)	SSI at time point: (1) on hospital discharge; (2) postoperative day 30 at hospital visit (superficial incisional/deep incisional/organ or space SSI), or by Bluebelle self-report wound healing validated survey^31^
Implementation outcomes		
Anticipated		
9	Implementability:appropriateness, acceptability, feasibility^32^	Pre and post implementation survey:(1) Acceptability of Intervention Measure; (2) Intervention Appropriateness Measure; (3) Feasibility of Intervention Measure^34^
10	Adoptability:intention to use^32^	Pre and post implementation survey:Adapted Clinicians’ Assessments of Practice Guidelines in Oncology tool^33^
11	Implementation readiness	Pre and post implementation survey:Organisational Readiness to Change^35^
Actual		
12	Implementation and sustainment: extent the simple principles and recommendations are being enacted^32^	Process of care audit of:Temperature monitoring: (1) preoperatively; (2) before induction; (3) intraoperatively; (4) in PACUActive warming: (1) preoperatively; (2) intraoperatively; (3) in PACU
13	Adoption: extent that teams decide to put the simple principles and recommendations in place^32^	Post implementation site survey
14	Fidelity of trial implementation strategy	Site implementation team meeting notes and activity log: trial implementation strategy, practice change initiatives, team attendance
15	Site implementation team experience and learning	Pre and post implementation survey:(1) Practicing knowledge translation questionnaire;^36^ (2) Implementation Climate Scale^37^Post implementation survey: Kirkpatrick’s levels of training association^38^
Economic outcomes		
16	Implementation costs	Cost of implementation activities: activity logs; clinical costing data; hospital bed days and admission costs (including equipment and consumable use); unplanned hospital readmissions within 28 days^40^
17	Cost-effectiveness	Quality-adjusted life years: EQ-5D-5L^39^ at: (1) preoperative baseline (within 48 hours prior to surgery); (2) postoperative day 3; (3) postoperative day 30

#### Effectiveness outcomes

The primary clinical effectiveness outcome is perioperative hypothermia, defined as a body temperature <36°C recorded within 15 min of post anaesthetic care unit (PACU) arrival time. Secondary clinical effectiveness outcomes include hypothermia at any perioperative time point, PACU and hospital length of stay, intraoperative or post-anaesthetic adverse events, blood transfusions and surgical site infection. Self-reported wound healing will be assessed at day 30 using the validated Bluebelle wound healing survey.^31^

#### Implementation outcomes

The primary implementation outcome is the extent of temperature monitoring and active warming, assessed by process of care audit. Secondary implementation outcomes include pre-transition measures of adoptability and implementability, and post-transition measures of adoption, fidelity of implementation strategy and site team learning. Adoptability (intention to use^32^) will be assessed via clinician and Site Implementation Team surveys survey using the Clinicians’ Assessments of Practice Guidelines in Oncology tool^33^ adapted for clinical context in our earlier work.^18^ Implementability (appropriateness, feasibility and acceptability^32^) of the simple principles and recommendations will be assessed during and post-implementation via these surveys using Weiner’s validated tools.^34^ We will survey adoption (the extent that teams decide to put the simple principles and recommendations in place^32^) via site level survey. Fidelity to the implementation strategy will be assessed via site meeting notes and activity. Context will be assessed pre-implementation and post-implementation via the clinician and Site Implementation Team surveys using the Organisational Readiness to Change 12-item instrument.^35^

Site Implementation Team learning and experience of participation will be assessed pre and post-transition period via surveys using the Practicing Knowledge Translation Questionnaire^36^ 20-item tool assessing knowledge and confidence in knowledge translation activities, the Implementation Climate Scale^37^ validated brief 18-item scale to measure climate for implementation. On completion of the transition period completion the survey will incorporate Kirkpatrick’s levels of training association^38^ to assess the impact of the implementation support.

#### Cost-effectiveness outcomes

Quality-adjusted life years (QALYs) will be assessed using the EQ-5D-5L^39^ at baseline (within 48 hours prior to surgery), at postoperative day 3 and postoperative day 30. Additional outcomes used for cost-effectiveness analysis include hospital bed days and admission costs (including equipment and consumable use), and unplanned hospital readmissions up to 28 days post-surgery (as per the Australian Commission for Safety and Quality in Health Care definition).^40^

### Data collection

#### Hospital data

For each month of the trial, a random sample of patient medical records, corresponding to 10% of monthly surgical cases (of between 59 and 226 health records), will be reviewed at each site currently in the control or intervention period. Random sampling will be stratified by duration of surgery (10%, ≤30 min; 30%, 30−60 min; 60%, ≥60 min). Outcome evaluators will extract data from patient records and hospital data sets (as outlined in table 1). Data will be recorded using structured data collection forms via REDCap (Research Electronic Data Capture) hosted on a secure drive. Patients will be assigned a unique study ID for de-identification and the purpose of linking data sets.

Hospital admitted data will include age (in years), sex, body mass index calculated by the most recent weight and height (within 3 months), comorbidities (using International Statistical Classification of Diseases, 10th Edition, Australian Modification referred to as ICD-10-AM classifications), American Society of Anesthesiologists’ (ASA) score, surgical specialty, mode of anaesthesia (general/spinal/epidural), urgency of surgery category and duration of surgery (in minutes from In Operating Room time to Out Operating Room time).

#### Patient-reported outcome surveys

Qualtrics will be used to distribute the quality of life tool (EQ-5D-5L^39^) at baseline, postoperative day 3 and postoperative day 30 via email or text message (as per the patient’s preference). On day 3, the follow-up survey will allow patients to indicate if their surgery did not proceed as planned, and the option to participate on their revised date of surgery. On day 30 the survey will also include the self-report wound infection survey,^31^ if patients indicate they have a wound.

#### Clinician and Site Implementation Team surveys

Electronic surveys and hard copy surveys will be distributed at each site pre-transition and post-transition period, with the assistance of departmental leads and assisted by site investigators.

#### Implementation activity logs

In collaboration with Site Implementation Teams, internal facilitators will maintain an activity log of all implementation activities including the number and role of participants, implementation strategy delivered and adaptations of planned strategy.

#### Participant follow-up

The subset of patients recruited to participate in data collection for PROs will be followed-up at 3 and 30 postoperative days for completion of the EQ-5D-5L^39^ and self-reported wound healing measures.^31^ For all other outcome measures from existing hospital data, data collection ceases at 30 days post-discharge for wound infection.

### Data analysis

#### Sample size determination and power

As recommended by Curran *et al*^41^ we have selected one co-primary aim in determining the required sample size: the clinical effectiveness outcome of perioperative hypothermia (table 1). The expected proportion of patients with perioperative hypothermia on PACU arrival at baseline is 0.274 (27%), based on a recent period prevalence study of five Australian hospitals.^2^ Minimum sample sizes were calculated based on the primary clinical outcome assuming unequal cluster sizes using a constrained randomisation approach.^29 30^ The use of constrained randomisation allows for site-specific sample sizes to represent a fixed proportion of their total surgical capacity, while aiming to minimise imbalance between the total sample sizes for control and intervention periods. For each month a site is in the control or intervention period, we will select a random sample of patients equal to 10% of monthly surgical cases, stratified according to the duration of surgery. Based on annual surgical numbers for participating sites, a 10% sample corresponds to 59–226 patients/site/month. To obtain at least 80% power, our proposed sampling strategy will detect a statistically significant and clinically relevant reduction from 0.274 (27%) in the control period to 0.2 (20%) in the intervention period. Calculations assume a 5% level of statistical significance, an expected intraclass correlation coefficient of 0.1. Across the constrained subset of possible randomisations, the total sample size for control and intervention periods combined will be between 12 732 and 14 100.

For the sample size, we have also considered the minimum detected increase in the primary implementation outcome. Given the lack of existing data on both monitoring and warming recommendations, we have used data on adherence to perioperative temperature monitoring at all recommended time points.^2^ Assuming that 2.7% of patients currently receive temperature monitoring at all recommended time points, with remaining parameters unchanged, we will be able to detect an increase from 0.027 (2.7%) in the control period to 0.1 (10%) in the intervention period with 82% power.

For the economic evaluation, a randomly selected subsample of up to 1500 patients will be directly recruited to complete PRO surveys, and prospectively followed-up (to allow for incomplete responses). This comprises an average of 300 patients per site over the 27-month trial duration. The sample size for the economic evaluation was determined based on the feasibility of directly recruiting participants with follow-up to 30 days post-surgery.

#### Primary analysis

The co-primary outcomes will be analysed using generalised linear mixed models (GLMM), assuming binary dependent variables. A random intercept will be defined for each site to account for clustering of observations under the stepped wedge design. Fixed effects will include intervention exposure (yes/no), calendar time to account for secular trends unrelated to the intervention (in months)^21^ and patient-level/surgery-level characteristics. The binary fixed effect for the intervention will estimate the expected within-site change in the co-primary outcomes associated with the intervention.

Associations between the dependent variable and fixed effects will be modelled using the logit link function. An identity link function will also be fitted as a comparison, to report risk differences. If the identity link function does not allow the model to converge, results for the logit link function will only be reported. Results for both unadjusted (except clustering) and adjusted analyses will be reported. Model parameters will be reported as estimated ORs (risk differences) with 95% CIs.

Subgroup analyses for the co-primary outcomes will examine intervention effects by sex, surgical specialty, type of anaesthesia, duration of surgery, elective/emergency, ASA classification I–V. A sensitivity analysis will also consider hypothermia defined at the following thresholds: <36°C, <35.5°C and <35°C.

#### Secondary analysis

Secondary outcomes will be similarly modelled based on the form of the dependent variable. Binary and count outcomes will be modelled using binomial and Poisson GLMMs, respectively. Models for count outcomes will assume a common denominator of cases per 1000 surgeries. Length of stay will be modelled using Cox proportional hazards regression. Analysis of survey and activity log data will be reported descriptively by study period.

Results for intention-to-treat (ie, all data analysed for all sites as randomised) and per-protocol (ie, data will be censored for sites if they withdraw from the trial protocol) will be reported. Missing data on primary and secondary outcomes is expected to be low. In the event of missing data on patient-level covariates, missingness will be investigated and if missing at random variables will be imputed using multiple imputation. Levels of missing data (if any) will be reported in planned research outputs.

#### Cost-effectiveness analysis

A within-trial cost-effectiveness analysis will compare costs and health outcomes of the evidence-based recommendations versus usual care from a health system perspective. The analysis will be conducted from the Australian health system perspective and the time horizon will be 30 days, consistent with the follow-up period of the trial. As such, discounting will not be required. Additional staff time involved with adoption will be estimated using activity logs completed by hospital staff and costed using mid-points of hospital salary ranges. Implementation costs including clinician engagement, and implementation activities will be prospectively recorded. Costs of equipment, materials and consumables, length of stay and readmissions will be retrospectively extracted from department budgets and hospital costing units. Hospital and staffing cost data will remain confidential.

The cost-effectiveness analysis will adopt QALYs as the measure of effectiveness.^39^ QALYs will be estimated using utility scores derived from the EQ-5D-5L questionnaire^39^ within the PRO subgroup across three time points. The EQ-5D-5L is the most widely used health utility tool internationally.^39^ QALYs will be derived by estimating the area under the curve using the methods described by Manca *et al*.^42^ Patients with only a baseline and no follow-up EQ-5D-5L^39^ questionnaires will be excluded from the cost-effectiveness analysis. For patients with one missing follow-up point, utility scores will be imputed using multiple imputation methods.

Results will be reported as incremental cost-effectiveness ratios (ICERs) and net monetary benefits using a willingness to pay threshold of $A50 000 per QALY. To assess uncertainty in the cost-effectiveness outcomes, a non-parametric probabilistic sensitivity analysis will be performed using bootstrapping of individual cost and QALY estimates with 10 000 replications. This will generate 10 000 ICERs which will be plotted on a cost-effectiveness plane and used to estimate mean and 95% CIs for net monetary benefit, along with cost-effectiveness acceptability curves. Where appropriate, one-way sensitivity analyses and scenario analyses will be conducted to assess the impact of key drivers on the cost-effectiveness outcome.

### Patient and public involvement

Patients and the public were not involved in the development of this study protocol. However, patients were involved in the development of the simple principles and recommendations,^18^ which will be evaluated in this trial. Plain language summaries of study findings will be developed for participants and wider patient communities to supplement planned publications.

## Ethics and dissemination

The study has obtained full ethical approval with waiver of consent from Metro North B Human Research Ethics Committee (reference: HREC/2023/MNHB/94571) and institutional administrative ethical approval. A waiver of consent was approved on the basis that the research carries no more than low risk to participants,^43^ the benefits from the research outweigh the risks of not seeking consent, it is impractical to seek consent due to the number of participants and records and there is sufficient protection of privacy and confidentiality.^43^ Site-specific, governance approvals have been obtained at each participating hospital. Queensland’s Public Health Act 2005 approval has also been granted for access to patient data. Any risk associated with access to data is minimised through the de-identification of data and ensuring data is accessed and stored according to the Australian Code for the Responsible Conduct of Research.^44^

Our dissemination plan includes strategies to support translation of findings into practice (where appropriate), as well as local, national and international dissemination across all relevant professional groups. If trial outcomes demonstrate benefit, we will disseminate the recommendations and implementation strategy to health services across Australia, drawing on the professional networks held by our large, interdisciplinary partnership team. The intent of this approach is to facilitate translation of results via diffusion, dissemination, implementation, thus promoting sustainability. For local dissemination at each hospital trial site, we will produce reports, presentations and plain language summaries. Our national and international dissemination includes submitting our findings for publication and presenting our work at relevant national and international health services conferences. Relevant reporting guidelines will be used in publications, as appropriate, including the Sex and Gender Equity in Research guidelines.^45^

## Discussion

Perioperative hypothermia prevention is complex. Our implementation design promotes coordinated action for hypothermia prevention across the surgical journey. Frontline teams will adapt implementation of guidance to suit their context. An external–internal facilitation model will support interdisciplinary team-based implementation at each site, guided by the KTA framework.^23^

Our effectiveness-implementation study will evaluate outcomes with a stepped-wedge cluster trial design. This allows all hospital sites to begin implementation at different times, with each site acting as its own control. By using hospital data collected for routine care, we maximise trial feasibility, but outcome evaluation relies on completeness of patient and hospital data sets. Some other potentially valuable outcomes of hypothermia prevention fall outside the scope of this trial, for example, patient-reported thermal comfort or shivering. These outcomes require prospective data collection methods that are not feasible within our trial design.

The GLOW trial is built on strong collaborative research partnerships between health services, universities and government organisations seeking to eliminate preventable perioperative hypothermia. It will contribute to our nascent understanding of how to bring about sustainable practice change for perioperative patient safety.
