# Supplementary material for: Implementing Guidelines for hypothermia prevention with Local adaptation to keep periOperative patients Warm (GLOW): protocol of a stepped-wedge cluster randomised hybrid type II effectiveness-implementation study
Source: BMJ Open. 2025 Feb 12;15(2):e091577. doi: 10.1136/bmjopen-2024-091577 (PMC11822429; doi:10.1136/bmjopen-2024-091577)
Supplement: Supplementary file 1 [file bmjopen-2024-091577.reviewer_comments.pdf]

PEER REVIEW HISTORY

BMJ Open publishes all reviews undertaken for accepted manuscripts. Reviewers are asked to complete a checklist review form and are provided with free text boxes to elaborate on their assessment. These free text comments are reproduced below.

ARTICLE DETAILS

Title (Provisional)

Implementing Guidelines for hypothermia prevention with Local adaptation to keep periOperative patients Warm (GLOW): Protocol of a stepped-wedge cluster randomised hybrid type II effectiveness-implementation study.

Authors

Munday, Judy; Duff, Jed; Wood, Fiona M; Sturgess, David; Keogh, Samantha; Ralph , Nicholas; White, Nicole Marie; Carter, Hannah; Graham, Ian D.

VERSION 1 - REVIEW

Reviewer 1

Name Pilkington, Mercedes

Affiliation SickKids Research Institute, Department of Pediatric Surgery

Date 26-Aug-2024

COI None

This is a very well written and well-designed randomized cluster hybrid type II effectiveness-implementation study that addresses the important topic of perioperative hypothermia. It is worthy of publication, and I anticipate with interest the results of applying consensus-based simple principles in place of implementing comprehensive guidelines.

A few minor comments or point of clarification:

1. Participants inclusion and eligibility: Please clarify if the subset of patients invited to participate in PROs are the same patients being recruited for the economic assessment. If not, please specify inclusion and eligibility for the economic assessment. If so, suggest describing a clarifying statement as it is unclear. Same comment regarding Recruitment and Sample Size Determination.

2. Screening of participants: this is the first mention of random sampling. Suggest describing in the 'Study design and setting' that only a random sampling of patients are being included and the justification (later described as 10% of monthly surgical cases).

3. Patient-reported outcomes survey: Can authors clarify why a sub-sample size of 1500 was selected for the economic evaluation?

4. Suggest describing the case mix at the 5 planned sites (e.g., inpatient/outpatient mix, any sub-specialization focus that may impact expected transfusion or surgical site infection rates).

5. In Table 1, please explain how is SSI being defined (i.e., does it include outpatient treatment for SSI?)? Are all patients expected to have a 30-day hospital visit?

6. Authors should consider adding a short discussion to include anticipated limitations

---

Reviewer **2**

Name **Pei, Lijian**

Affiliation **Peking Union Medical College Hospital, Department of Anaesthesiology**

Date **07-Sep-2024**

COI **none**

This study is the first large-scale effectiveness-implementation study to evaluate consensus-based principles and team-based local adaptation for perioperative hypothermia prevention targeted 36.0. It focuses on practical application by using simple principles and recommendations, allowing teams to tailor improvement strategies to their specific settings.

Stepped-Wedge Cluster Randomized Design: This design allows all hospital sites to begin implementation at different times, with each site acting as its own control, reducing bias and enhancing statistical power.

External-Internal Facilitation Model: Guided by the Knowledge-to-Action framework, this model supports interdisciplinary team-based implementation, effectively driving change.

However, I have several concerns regarding this study protocol:

Why is the target final core temperature set at 36°C instead of 35.5°C, given that the large-scale PROTECT trial showed similar major complication rates between 35.5°C and 37°C?

On page 11, Table 1: for outcomes 4 and 5, the length of stay in the PACU is often influenced by human factors. Would it be more objective to use the time to extubation instead? Additionally, for intraoperative events: how are "complications during the surgical episode" defined?

In the health economic analysis, how will the cost information and personnel service data be obtained from each hospital? Will this information remain confidential?

These strengths highlight the study's innovative approach, feasibility, and practical application, while the limitations point to challenges in data collection.

VERSION 1 - AUTHOR RESPONSE

**Reviewer 1**		
**Comments to the Author:**<br>This is a very well written and well-designed randomized cluster hybrid type II effectiveness-implementation study that addresses the important topic of perioperative hypothermia. It is worthy of publication, and I anticipate with interest the results of applying consensus-based simple principles in place of implementing comprehensive guidelines.	Thank you for your considered review of our manuscript.	
**A few minor comments or point of clarification:**<br>1. Participants inclusion and eligibility: Please clarify if the subset of patients invited to participate in PROs are the same patients being recruited for the economic assessment. If not, please specify inclusion and eligibility for the economic assessment. If so, suggest describing a clarifying statement as it is unclear. Same comment regarding Recruitment and Sample Size Determination.	Yes, the subset of patients invited to participate in PRO surveys are the same patients included in the economic analysis. As per your suggestion, we have clarified this in the Participant Inclusion and Eligibility Criteria, Recruitment and Sample Size Determination sections.	Page 7, paras 2, 4.<br>Page 13, para 3.
2. Screening of participants: this is the first mention of random sampling. Suggest describing in the 'Study design and setting' that only a random sampling of patients are being included and the justification (later described as 10% of monthly surgical cases).	We have added that a random sample of patients will be included from each hospital in the *Study design and setting* section, as suggested.	Page 5, para 2.
3. Patient-reported outcomes survey: Can authors clarify why a sub-sample size of 1500 was	We have added explanation that the sub-sample of 1,500 was determined based upon the feasibility of directly recruiting patients and	Page 13, para 3.

selected for the economic evaluation?	follow-up over three timepoints. Our economic evaluation is focused on estimation rather than hypothesis-testing and so will provide useful information about costs and benefits of hypothermia prevention.	
4. Suggest describing the case mix at the 5 planned sites (e.g., inpatient/outpatient mix, any sub-specialization focus that may impact expected transfusion or surgical site infection rates).	We have added more detail about the case mix across sites. We used a constrained randomisation approach accounting for size of hospital and with a stepped wedge cluster trial, each hospital acts as its own control.	Page 5, para 2.
5. In Table 1, please explain how is SSI being defined (i.e., does it include outpatient treatment for SSI?)? Are all patients expected to have a 30-day hospital visit?	The definitions of SSI have been added to Table 1. SSI data is extracted from existing Infection Control hospital datasets – patients are not expected to have a 30-day hospital visit.	Table 1, page 11
6. Authors should consider adding a short discussion to include anticipated limitations	We have added a short discussion with anticipated limitations as suggested.	Page 16
**Reviewer 2**		
**Comments to the Author:**<br>This study is the first large-scale effectiveness-implementation study to evaluate consensus-based principles and team-based local adaptation for perioperative hypothermia prevention targeted 36.0. It focuses on practical application by using simple principles and recommendations, allowing teams to tailor improvement strategies to their specific settings.<br>Stepped-Wedge Cluster Randomized Design: This design allows all hospital sites to begin implementation at different times, with each site acting as its own control, reducing bias and enhancing statistical power.<br>External-Internal Facilitation Model: Guided by the Knowledge-to-Action framework, this model supports interdisciplinary team-based implementation, effectively driving change.	Thank you for your review of our manuscript.	

However, I have several concerns regarding this study protocol:<br>Why is the target final core temperature set at 36°C instead of 35.5°C, given that the large-scale PROTECT trial showed similar major complication rates between 35.5°C and 37°C?	Thank you for the question. Our primary outcome uses a definition of hypothermia (<36°C) which aligns with our published national consensus on perioperative hypothermia: this is the evidence that is being implemented. Acknowledging your question, our sensitivity analysis will also consider hypothermia at several thresholds of severity (including <35.5°C and <35°C) (please see page 13).	
On page 11, Table 1: for outcomes 4 and 5, the length of stay in the PACU is often influenced by human factors. Would it be more objective to use the time to extubation instead? Additionally, for intraoperative events: how are "complications during the surgical episode" defined?	Thank you for the suggestion. In the Australian context, extubation most often occurs prior to PACU admission. Our hypothesis is that hypothermia is a significant contributor to delays in PACU discharge to the ward. Patient temperature status / hypothermia commonly forms part of PACU discharge criteria.<br><br>Intraoperative events are extracted from complications recorded and selected from predefined event lists within hospital operating room electronic records.	
In the health economic analysis, how will the cost information and personnel service data be obtained from each hospital? Will this information remain confidential?	Comment added about ensuring confidentiality. We have checked that data sources are described in the manuscript and Table.	Page 14, para 3
